# 

**Published:** 1960-10

**Authors:** 


					Contents
^CTC^o^ETICS
AND THE
Thomas E. Oppe
77
Leslie Hill
89
The
evolution of paediatrics
John Apley
98
^?nS?INGEAL
Clinical Pathological Conference
i?5
PROM SOCIETIES
io8
tments

				

